# Dorset Pre-Inuit and Beothuk foodways in Newfoundland, ca. AD 500-1829

**DOI:** 10.1371/journal.pone.0210187

**Published:** 2019-01-07

**Authors:** Alison J. T. Harris, Ana T. Duggan, Stephanie Marciniak, Ingeborg Marshall, Benjamin T. Fuller, John Southon, Hendrik N. Poinar, Vaughan Grimes

**Affiliations:** 1 Department of Archaeology, Memorial University of Newfoundland, St. John’s, Newfoundland, Canada; 2 McMaster Ancient DNA Centre, Department of Anthropology, McMaster University, Hamilton, Ontario, Canada; 3 Institute of Social and Economic Research, Memorial University of Newfoundland, St. John’s, Newfoundland, Canada; 4 Keck Carbon Cycle Accelerator Mass Spectrometry Laboratory, Earth Systems Science Department, University of California Irvine, Irvine, California, United States of America; 5 Michael D. DeGroote Institute for Infectious Disease Research, McMaster University, Hamilton, Ontario, Canada; 6 Humans & the Microbiome Program, Canadian Institute for Advanced Research, Toronto, Ontario, Canada; 7 Department of Earth Sciences, Memorial University of Newfoundland, St. John’s, Newfoundland, Canada; Institucio Catalana de Recerca i Estudis Avancats, SPAIN

## Abstract

Archaeological research on the Canadian island of Newfoundland increasingly demonstrates that the island’s subarctic climate and paucity of terrestrial food resources did not restrict past Pre-Inuit (Dorset) and Native American (Beothuk) hunter-gatherer populations to a single subsistence pattern. This study first sought to characterize hunter-gatherer diets over the past 1500 years; and second, to assess the impact of European colonization on Beothuk lifeways by comparing the bone chemistry of Beothuk skeletal remains before and after the intensification of European settlement in the early 18^th^ century. We employed radiocarbon dating and stable carbon and nitrogen isotope ratio analysis of bulk bone collagen from both Dorset (n = 9) and Beothuk (n = 13) cultures, including a naturally mummified 17^th^ century Beothuk individual. Carbon and nitrogen isotope analysis of 108 faunal samples from Dorset and Beothuk archaeological sites around the island were used as a dietary baseline for the humans. We combined our results with previously published isotope data and radiocarbon dates from Dorset (n = 12) and Beothuk (n = 18) individuals and conducted a palaeodietary analysis using Bayesian modelling, cluster analysis and comparative statistical tests. Dorset diets featured more marine protein than those of the Beothuk, and the diets of Beothuk after the 18^th^ century featured less high trophic level marine protein than those of individuals predating the 18^th^ century. Despite inhabiting the same island, Dorset and Beothuk cultures employed markedly different dietary strategies, consistent with interpretations of other archaeological data. Significantly, European colonization had a profound effect on Beothuk lifeways, as in response to the increasing European presence on the coast, the Beothuk relied more extensively on the limited resources of the island’s boreal forests and rivers.

## Introduction

The addition of stable isotope ratio analysis to the study of coastal hunter-gatherer lifeways has garnered fresh evidence for subsistence and land use patterns, and has validated research hypotheses informed by ethnographic and ethnohistoric sources [1]. Bioarchaeological studies employing carbon (δ^13^C) and nitrogen (δ^15^N) isotope ratio analysis of human bone collagen and hair from Arctic-adapted populations in North America have highlighted diachronic and geographic variation in human diet, and revealed both the development of marine adaptations, and the dynamics of human-animal interactions [2–6]. The Canadian province of Newfoundland and Labrador has been home to human populations for up to 8000 years [7,8]. Skeletal remains affiliated with Native American and Pre-Inuit cultures have been recovered from the island of Newfoundland and analysed from a morphometric perspective [9,10], but stable isotope-based approaches have been underutilized in the study of human subsistence on the island. Previous ancient DNA studies of the Pre-Inuit Dorset population and Beothuk people have produced stable isotope data for the purpose of calibrating radiocarbon dates, but the potential of the isotope data for investigating past subsistence practices on the island has not yet been thoroughly explored [11,12]. This is unfortunate as the boreal ecology of the island and the archaeological evidence for human subsistence make it an ideal context for the use of stable isotope ratio analysis to investigate the relative contributions of marine and terrestrial foods to hunter-gatherer diets. Recent archaeological research has revealed that the island’s social environment was complex and hunter-gatherer groups may have negotiated cross-cultural interactions by employing specific land-use strategies in both the pre- and post-European contact periods [13–15], a line of inquiry that a bioarchaeological approach is well situated to address.

We conducted stable isotope analysis, radiocarbon dating, and ancient DNA analysis on the remains of 159 individuals in order to examine the biocultural relationships of Newfoundland’s past indigenous populations, and to provide crucial chronological information needed to interpret cultural development and change over the past 8000 years of Newfoundland and Labrador prehistory. Here we present 17 new radiocarbon dates and stable isotope data from the skeletal remains of 22 individuals affiliated with the Beothuk and Dorset cultures. We also sampled multiple skeletal elements of two Beothuk individuals to investigate the possibility of dietary change over the life course. The isotope data are interpreted with reference to the published datasets of [7, 11,12], and through comparison with previously unpublished carbon and nitrogen isotope data from the remains of 108 local archaeological fauna. The data support current interpretations of the archaeological record and indicate that the differences in Dorset and Beothuk site distribution clearly relate to different hunting and dietary practices. We find compelling new evidence for a change in Beothuk lifeways in relation to the island’s expanding European population in the 18^th^ century.

### Archaeological and environmental context

**Fig 1:**
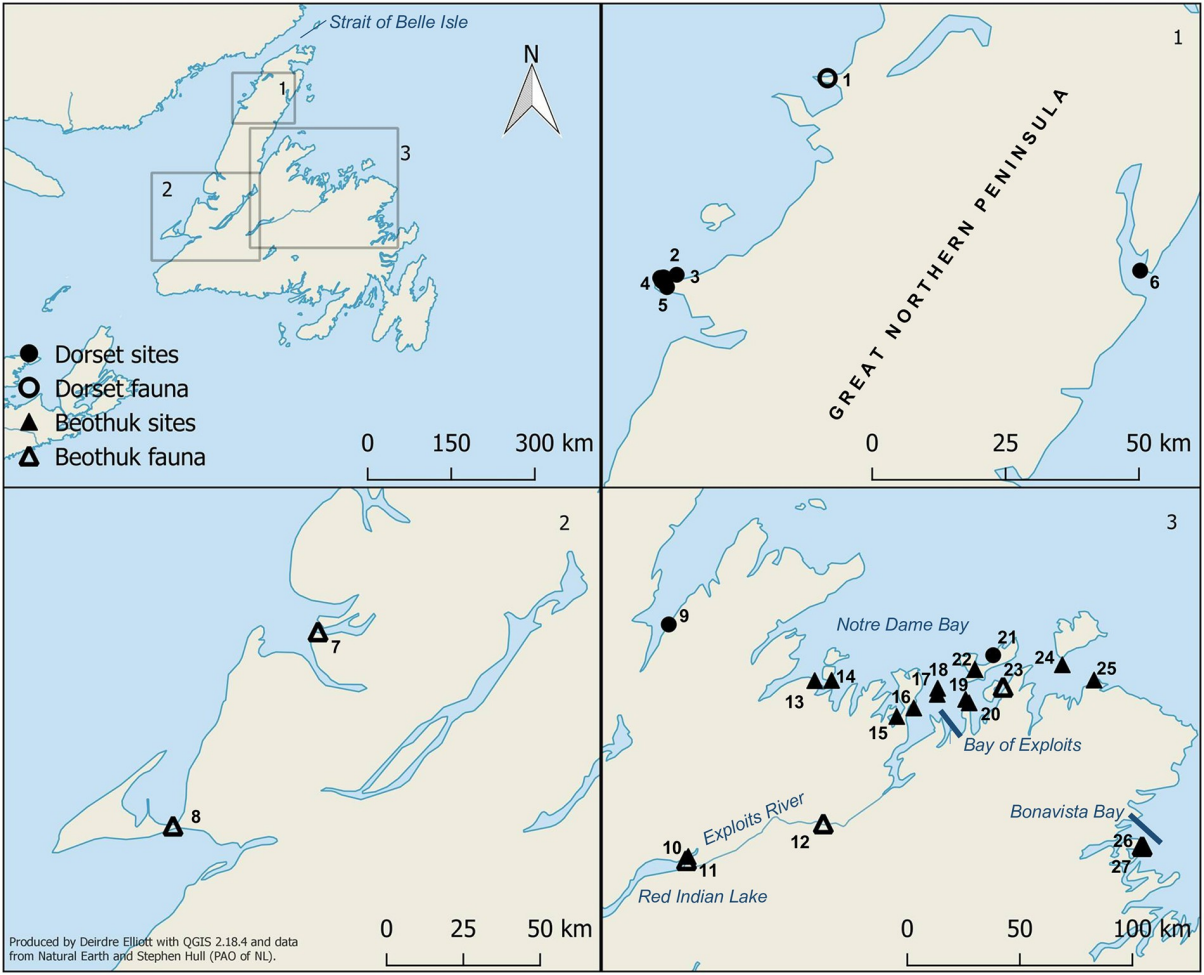
Location of sites mentioned in the text. 1) Peat Garden North; 2) Phillip’s Garden; 3) Crow Head Cave; 4) Eastern Point; 5) Gargamelle Rockshelter; 6) Lane’s Cove/Englee; 7) Parke’ Beach; 8) Port au Port; 9) Pumbley Cove; 10) Red Indian Lake; 11) Indian Point; 12) North Angle; 13) Big Island; 14) Devil’s Cove; 15) Long Island; 16) High Greco Island; 17) Charles Arm; 18) Swan Island; 19) Comfort Island; 20) Cranberry Island; 21) Rogers Cove; 22) Indian Cove; 23) Boyd’s Cove; 24) Western Indian Island; 25) Ladle Point; 26) Fox Bar; 27) Beaches.

The island of Newfoundland (Fig 1) is located in the North Atlantic and is separated from the mainland of Quebec and Labrador to the north by the Strait of Belle Isle, which ranges in width from 15 to 65km. The island’s vegetation is characterized by subarctic scrub forest and peatlands on the Great Northern Peninsula to a mixed boreal forest along the north and east coasts [16]. Archaeological research since the late 1990s has revealed that over the past 2000 years, Newfoundland’s hunter-gatherers occupied a complex social arena [13,15]. Archaeological traces of two culturally distinct groups, represented by a Pre-Inuit population known archaeologically as the Dorset culture, and pre-contact Beothuk archaeological complexes, have been found on the island and date to 2110 and 2100 cal B.P., respectively [17,18]. The origin of the Dorset can likely be found in an earlier Pre-Dorset culture that spread across the Arctic from Siberia 4000 years ago [19,20] while the Beothuk were related to other indigenous North American populations [7]. Different subsistence patterns and material culture have been attributed to each of these cultural groups based on archaeological and ethnohistoric evidence [21,22].

The Beothuk were among the first indigenous populations in North America to experience sustained European contact, but the tenor of their interactions with French and English migratory fishermen differed significantly from those experienced by indigenous people in the other Atlantic Provinces of Canada [23]. Their population size at European contact has been estimated at 500 to 1000 individuals, but this number may have fallen to 350 by the mid-18^th^ century [24]. Contact between the Beothuk and European fishermen was limited and, despite some initial attempts, a formal trading partnership never developed [25,26]. European economic activities were focused upon the migratory cod fishery, and in the 16^th^ and early 17^th^ centuries little effort was invested in building and maintaining infrastructure beyond the construction of wooden stages on the shoreline for fish processing [27]. By the onset of the 19^th^ century, European settlement had intensified and as settlers expanded further into the traditional territories of the Beothuk along the northeastern shore, contact became more frequent between the two groups [24]. The nature of the relationship was unpredictable and frequently antagonistic as fishermen and Beothuk clashed over access to productive salmon fishing rivers [24,28]. To mitigate the danger posed by European fishermen, the Beothuk retreated from their coastal settlements and established themselves in the interior, particularly along the Exploits River [24]. Away from the productive coastlines, Newfoundland’s interior is subsistence resource poor, but the Beothuk may have coped for a time by intensifying their reliance on herds of woodland caribou (*Rangifer tarandus*). Ethnohistoric accounts refer to long caribou fences and wooden storehouses filled with caribou meat, while the location of Beothuk sites on the north and south banks of the Exploits River may evidence Beothuk efforts to intercept caribou during both spring and fall migrations [28,29]. As their population dwindled, it became increasingly difficult to maintain the fenceworks which enabled survival in the interior and the Beothuk are reported to have suffered from deprivation and hunger in the years prior to their cultural extinction [24]. It is largely accepted that, in addition to violent altercations and increased exposure to disease, the changes in Beothuk subsistence in the 18^th^ and early 19^th^ century contributed to the collapse of their population [28,30]. By the onset of the 19^th^ century, Beothuk population numbers had declined precipitously and in AD 1829, Shanawdithit, thought to be the last Beothuk woman, died in St. John’s, NL [29].

For much of the 20^th^ century, pre- and post-European contact hunter-gatherer foodways in Newfoundland have been understood through ethnographic study of the present indigenous peoples of mainland Labrador and Quebec [31]. It was assumed, that like the Innu to the north, the Beothuk balanced spring and summer marine hunting with an autumn caribou hunt and a winter reliance on small game [31]. Archaeological research has produced more evidence of both Beothuk and earlier Dorset lifeways, but the data is biased by the selective focus on coastal sites, and variable preservation affecting organic remains [17,32]. These data, combined with detailed analyses of the geographic distribution of sites now evidence widespread marine adaptations across all pre-contact indigenous groups, albeit with variations occurring in the prey classes targeted, tool forms and settlement patterns. Specialized Dorset seal hunters accessed migrating herds of seals by locating their sites along the exposed outer coasts of the island, while Beothuk communities adopted a generalized marine strategy and occupied the protected inner shore regions and deep bays where the movements of caribou and a variety of marine resources could be monitored [14,21,22,33,34]. However, questions still remain as to 1) the degree of diachronic variation in Beothuk lifeways, particularly after the 18^th^ century; and 2) seasonality and the role of inland terrestrial resources in Dorset and Beothuk subsistence practices.

### Isotopic analysis and hunter-gatherer diets in Newfoundland

We employed δ^13^C and δ^15^N analysis of human and faunal bone collagen to evaluate the relationship between culturally specific settlement patterns and the diets of Dorset and Beothuk people over the past 2000 years. The isotopic composition of bone collagen is directly related to diet as the chemical elements from consumed food become assimilated into bone and dental collagen during growth and turnover [35]. The δ^13^C value of collagen largely reflects the isotopic composition of dietary protein, but some input from whole dietary carbon (e.g. carbohydrates and lipids) can be expected depending on the amount and quality of protein in the diet [35–37]. A trophic shift of approximately +5‰ has been shown to occur between diet and consumer tissues [38], however, the isotopic shift between prey and consumer bone collagen is significantly smaller, ranging from <+1‰ to +2‰ [39], which enables the use of δ^13^C values to track the average sources of consumed dietary protein (e.g. marine or terrestrial). Bulk collagen δ^15^N values also track protein sources but are elevated relative to dietary protein. Published diet-tissue offsets (Δ^15^N) range from +2‰ to +6‰ [40–42], but an average of +3‰ to +5‰ is often used to interpret foodweb trophic dynamics in archaeology [43]. The δ^13^C and δ^15^N values measured in collagen are generally understood to reflect upwards of 20 years of diet due to the slow rate of collagen turnover in the bones of healthy adults [44]. Meanwhile, the isotopic composition of dentinal collagen reflects only the foods ingested during the period of dentine growth which occurs between infancy and adolescence and is dependent upon the sampled tooth [45].

Marine and C_3_ terrestrial protein-based diets produce distinguishable δ^13^C and δ^15^N values in consumer tissues [46,47]. Due to isotopic fractionation associated with the C_3_ photosynthetic cycle, plants growing in the boreal and subarctic zones of Newfoundland should have δ^13^C values averaging -28‰ [48,49]. Marine autotrophs obtain carbon from marine bicarbonate, and dissolved CO_2_, and have δ^13^C values of approximately -21‰ [39]. Cumulative enrichment in ^15^N occurs between trophic positions; in northern regions, the highest δ^15^N values are observed in the long food chains of aquatic environments [50]. The isotopic differences between marine and terrestrial consumers are frequently used to investigate marine adaptations in past cultures around the globe [2], and have been particularly useful for investigating the influence of European settlement on indigenous lifeways in the Americas [1,51,52].

The δ^13^C and δ^15^N analysis of 12 Dorset individuals from the sites of Gargamelle Rockshelter, Phillip’s Garden, Eastern Point, Indian Cove, and Lane’s Cove/Englee (Fig 1) on Newfoundland’s Great Northern Peninsula produced high δ^13^C and δ^15^N values that were consistent with a specialized adaptation targeting marine mammals [7,12]. We expected that the isotopic composition of an additional two adults and seven subadults from the sites of Crow Head Cave and Lane’s Cove/Englee (Fig 1) would show a similar pattern. Based on the available archaeological and ethnographic evidence, we expected the majority of the Beothuk individuals to occupy a lower trophic level than the Dorset, and we further hypothesized that Beothuk individuals postdating the 18^th^ century would display a noticeable decrease in δ^13^C and δ^15^N values relating to increased consumption of terrestrial protein. Some indication of this chronological trend was apparent in the data presented by [7,11], but a larger sample size is required to distinguish potential regional and/or temporal differences. As the aforementioned studies focused on ancient DNA analysis of these populations, the isotopic data were not previously modelled with respect to a local faunal baseline, limiting the interpretation of the dietary data. Therefore, we compare these previous data sets with unpublished carbon and nitrogen isotope data from the collagen of 22 human skeletal remains and 108 faunal specimens to address the following questions: 1) Do the stable isotope data from Dorset and Beothuk human remains support the hypothesis that each cultural group had distinct foodways and settlement patterns?; and 2) Does the bioarchaeological record reveal an increasing reliance on terrestrial sources of protein among the Beothuk after the early18^th^ century?

## Materials and methods

### Human samples

The total number of human skeletal remains included in our palaeodietary analysis were recovered from 19 archaeological sites on the island of Newfoundland, listed in Table 1. Dorset mortuary traditions are difficult to define in the Eastern Arctic and Subarctic [53] and here we have based our cultural designation upon associated material culture, radiocarbon dates that are consistent with the Dorset occupation of Newfoundland, geographic location, and mtDNA sequences, where available. Published mtDNA sequences from individuals from the sites of Phillip’s Garden, Gargamelle Rockshelter, Lane’s Cove/Englee, and Eastern point have been used to characterize the Pre-Inuit, including the Dorset, genetic profile [7,12], and as such, we are confident in the cultural affiliation of these individuals. Our study sample includes two sites of uncertain, but likely Dorset context: Indian Cove in the Bay of Exploits, and Englee. The Indian Cove site (DjAq-15) was discovered during an archaeological survey in 1974. Stone tools manufactured in the Dorset tradition and faunal remains were recovered, but never officially reported (S. Hull, Provincial Archaeology Office, Personal communication, 2015). In 2006 human skeletal elements were noted amidst the faunal remains and were transferred to the osteology laboratory of the Department of Archaeology at Memorial University (MUN). Osteological analysis suggested a minimum number of two individuals, however, the radiocarbon dates and isotopic data suggested that only a single individual was present. Later mtDNA analysis identified two distinct mitochrondrial haplotypes confirming the initial assessment of two skeletons: one (D2a1) found in ancient Pre-Inuit skeletons, and the other (A2i) in Native American cultures, including the Beothuk [7].

**Table 1:** Summary of archaeological contexts of the human skeletal remains analysed in this study.

Site	Borden No.	Specimen	Time Period	Year of collection	Reference
The Rooms Corp.					
Big Island	DjAw-17	NP 240	Post-contact Beothuk	1888	Patterson (1892), Howley (1915)
Big Island	DjAw-18	NP 265	Post-contact Beothuk	1888	Patterson (1892), Howley (1915)
Comfort Island	DiAr-01	NP 152 NP 294 NP 296	Post-contact Beothuk	1888	Marshall (1996)
Cranberry Island	DiAr-08	NP 151	Post-contact Beothuk	1973/74	Marshall (1984)
Crow Head Cave	EeBi-04	NP 55 NP 55B NP 55C NP 55E	Dorset	1986	Brown (2011)
Devil’s Cove	DjAw-16	NP 299	Pre-contact Beothuk	1973	LeBlanc (1973)
Eastern Point	EeBi-35	NP 174	Dorset	1904	Howley (1915)
Englee	-	NP 70A NP 70B NP 70C NP 70D NP 70E NP 70(2)	Dorset	1972	Tuck n.d.
Fox Bar	DeAK-02	NP 270A NP 270B NP 270C NP 270D NP 270E NP 270F NP 270G	Proto-contact/Post-contact Beothuk	1973	Carignan (1973)
Gargamelle Rockshelter	EeBi-21	NP 172_1 NP 172_2 NP 172_3 NP 172_4 NP 172_5	Dorset	1953–1986	Harp & Hughes (1968), Brown (2011)
High Greco Island	DiAt-03	NP 290	Post-contact Beothuk	Ca. 1935	Devereux (1965)
Indian Cove	DjAq-15	NP 297 NP 298	Dorset or Pre-contact Beothuk	1974	No reference
Ladle Point	DiAm-01	NP 266	Post-contact Beothuk	1834	Howley (1915)
Lane’s Cove/Englee	EeBa-01	NP 57	Dorset	Late 1960s	Anderson & Tuck (1974)
Phillip’s Garden	EeBi-01	NP 173B	Dorset	-	Harp & Hughes (1968)
Pumbley Cove	DkBe-04	NP 56	Dorset	1959	Anderson & Tuck (1974)
Rogers Cove	DjAp-06	NP 268	Post-contact Beothuk	1997	No reference
Swan Island	DiAs-09	NP 291 NP 292	Post-contact Beothuk	1886	Howley (1915)
Canadian Museum of History					
Charles Arm	DiAt-02	XIII-A-13a XIII-A-13b XIII-A-14	Post-contact Beothuk	1965	Devereux (1969)
Devil’s Cove	DjAw-16	XIII-A-12	Post-contact Beothuk	1973	LeBlanc (1973)
Long Island	DiAs-06	XIII-A-1 XIII-A-2-1 XIII-A-2-2	Post-contact Beothuk	1927	Jenness (1929)
Swan Island	DiAs-09	XIII-A-10	Post-contact Beothuk	1886	Howley (1915)
Western Indian Island	DjAn-01	XIII-A/3c	Post-contact Beothuk	1948	Marshall (1996)

The associated chronological/cultural period is defined by radiocarbon dates, where available, and recovered artifacts. The specimens are separated by the location of permanent repository.

The last Dorset site in our study is that of Englee (Fig 1, site 6). There is some uncertainty as to the number of sites to be included under the designation of Englee. In the late 1960s, the articulated remains of a single subadult were recovered with a slate projectile point from a rock crevice in Lane’s Cove which subsequently received the site designation of EeBa-1 [9]. A mtDNA sequence was later obtained from this individual that was consistent with Pre-Inuit populations [12]. In the early 1970s, construction workers discovered the remains of up to six individuals, and numerous grave offerings [54]. The human remains were consigned to the MUN osteology laboratory under the designation of “NP 70 –Englee”. The material culture from the site consisted of typical Dorset artifacts, such as toggling harpoon parts, a stone lamp, and zoomorphic amulets, as well as unusual artifacts identified as whale bone lances [54]. A mtDNA sequence from one individual from the site is consistent with other Pre-Inuit individuals [7]. Despite these data, the site does not appear to have obtained its own Borden number, or recognition in the archaeological literature.

The 34 Beothuk samples included in our analysis, representing 31 individuals, were obtained from 13 sites, and of these, 12 are deemed to be authentic Beothuk mortuary sites on the basis of grave offerings, burial location, and the inclusion of particular mortuary elements, such as red ochre or birch bark [24]. The Beothuk consigned their dead to rock shelters and caves along the Newfoundland coast, with clusters of burials found in Notre Dame Bay and the Bay of Exploits on Newfoundland’s northeastern shore. Grave offerings are typically utilitarian and feature tools of both Beothuk and European make, and faunal remains, but items that may have held spiritual significance have also been found, the most notable of which are carved and incised bone and ivory ornaments that have been recovered, sometimes in large numbers, from at least 10 Beothuk burial sites [55]. One individual is tentatively identified as Beothuk, even though the burial context is inconsistent with Beothuk mortuary tradition. The individual from Roger’s Cove (NP 268), represented only by a cranium, was recovered by the Royal Canadian Mounted Police from a secondary context on Fogo Island. This individual was likely affiliated with the Beothuk culture based on the general location of the find (Fig 1, site 21) [56], a radiocarbon date that indicates the individual died during the late 16^th^ to early 17^th^ century, isotopic values consistent with a marine protein-based diet, and a mtDNA haplotype [7] consistent with those sequenced to date in ancient Native Americans [57]. Further details on the archaeological context of each Beothuk site can be found in [7].

The palaeodietary study includes 14 subadult individuals: eight from Dorset and six from Beothuk contexts. However, given the commingled nature of the sites, only one could be aged with confidence, while the others have received the designation of ‘subadult’ based on the small size of the sampled skeletal elements. Between 1882 and 1886, the naturally mummified remains of a juvenile (NP 240) were collected from the site of Big Island (Fig 1, site 13) [29]. The child, approximately four years of age, was found wrapped in an article of hide clothing [58] with parcels of dried food, a miniature canoe and a human figurine, among other artifacts [29]. The partial skeletal remains of an adult (NP 265) and several artifacts of European origin were found nearby [24]. A sample of soft tissue from the child was radiocarbon dated by [59] and returned a date of 549 +/- 63 years BP. Despite the fragmentary nature of the Beothuk and Dorset skeletal assemblages, all other individuals, including those who could not be aged reliably and are thus given the age designation of ‘unknown’, are assumed to have reached adulthood, or at least adolescence, at the time of death based on the size and morphology of the sampled skeletal elements.

### Faunal samples

In order to interpret stable isotope ratio data from human collagen, the δ^13^C and δ^15^N values were referenced to an isotopic baseline developed from contemporaneous, local faunal bones [60,61]. We included faunal samples from two Dorset sites located on the west coast of the Great Northern Peninsula: the mortuary site of Crow Head Cave (EeBi-4), and the habitation site of Peat Garden North (EgBf-18); we also sampled faunal remains from five Beothuk sites located on the northeastern coast of the island, the west coast, and the interior of the island along the Exploits River.

### Permits and repository information

All necessary ethics approvals and permits were obtained for the following study, which complied with all relevant regulations. The human remains are held in permanent repositories at the Canadian Museum of History (Ottawa, Canada) and in the Rooms Corp. Provincial Museum (St. John’s, Canada). We received written permission for destructive analysis of human remains from the local indigenous communities of Newfoundland and Labrador, the Rooms Corp. Provincial Museum Division, and by the Canadian Museum of History. The Rooms Corp. Provincial Museum Division granted permission for the destructive analysis of faunal bone specimens under the Loan No. L-2015-16. The faunal samples do not have individual specimen numbers, however, the associated laboratory numbers, and archaeological site borden numbers can be found in S4 Table. Neither the human or faunal specimens are available to the public but can be accessed upon written request.

### Collagen extraction and stable isotope analysis

Human and faunal bone collagen samples were prepared for δ^13^C and δ^15^N analysis at the Memorial Applied Archaeological Sciences (MAAS) laboratory using a modified Longin method [62,63] with a sodium hydroxide (NaOH) pretreatment modified from [64]. Bone samples were taken from the ribs or long bones of the 22 individuals not included in previous studies, and from 108 faunal specimens. Small pieces of bone, ~150 to 200 mg in mass, were removed using a handheld rotary tool (Jobmate) and cleaned of debris and cancellous bone using air abrasion with aluminium oxide powder. The bone samples were demineralized in chilled 0.5M hydrochloric acid (HCl) and those that appeared to be contaminated with humic substances were ultrasonicated in ~10 ml of 0.025M NaOH (pH 12.4) in an ice bath following demineralization. The NaOH solution was refreshed every ten minutes until the solution remained clear and colourless, after which time the samples were removed from the NaOH solution, rinsed with 0.1M HCl, followed by three rinses with deionized water. The samples were gelatinized in a dilute HCl solution (pH 3) on a heater block (70°C, 48 hours) to produce a collagen residue. The residues were filtered with E-zee filters (Elkay, UK) to remove particulate contaminants, followed by ultrafiltration (30kD, Pall Corporation) for samples that appeared poorly preserved (friable or darkly stained). The filtered collagen samples were frozen and lyophilized for 48 hours.

Isotopes of carbon and nitrogen were measured at the TERRA Stable Isotope Laboratory of the CREAIT Network at Memorial University of Newfoundland. Collagen samples (1 mg) were weighed into tin capsules (7x7 ultralight, Elemental Microanalysis, Southampton, UK), tightly folded and flash combusted at 1800°C in a Carlo Erba NA 1500 Series elemental analyser. The resulting CO_2_ and N_2_ gases were introduced to a Delta V Plus mass spectrometer via a ConFloII interface. Isotope ratios were calculated according to the equation δX(‰) = [(R_sample_/R_standard_)-1] × 1000 where X equals the isotope ratio of interest (^13^C/^12^C or ^15^N/^14^N), and R the isotope ratio of either the sample or a standard of known isotopic composition. The isotopic compositions were calibrated to the VPDB and AIR scales using D-Fructose, IAEA-N-2, and EDTA #2. Analytical accuracy was 0.23‰ and 0.03‰ for δ^13^C and δ^15^N measurements, respectively, as measured using a check casein standard, B-2155. Analytical precision on replicate collagen samples was 0.13‰ and 0.10‰ for δ^13^C and δ^15^N measurements, respectively. Further details of the standards, such as their isotopic and elemental composition, can be found in S1 Table. The preservation of human and faunal bone collagen was monitored using the atomic ratio of carbon to nitrogen (C:N) and the weight percent of carbon (%C) and nitrogen (%N) measured in each sample. Samples deemed acceptable had C:N ratios between 3.0 and 3.5, and %C and %N greater than 30% and 11%, respectively [65].

### Radiocarbon dating and calibration

All of the radiocarbon dates from human bone collagen reported here were produced by the Keck Carbon Cycle AMS Facility at the University of California Irvine. To correct for the marine radiocarbon reservoir effect, we calculated the percentage of marine carbon in each sample by interpolating the sample δ^13^C value between two endpoints determined via analysis of marine and terrestrial faunal bone collagen. Further details can be found in [7]. We calibrated the radiocarbon dates using OxCal v. 4.3 [66] using mixed IntCal13 and Marine13 [67] curves according to the percent marine carbon calculation with an estimated uncertainty of 10% [68]. We applied a ΔR correction of 140 ± 50 years, after [12] and [7]. While this ΔR was deemed to be appropriate for marine-adapted populations from the Great Northern Peninsula, it may not be appropriate for the Beothuk people who primarily occupied the northeastern coast of Newfoundland. This region is characterized by small islands that protect the coastline, in addition to an influx of freshwater from the Exploits and Gander Rivers, therefore, it is possible that differences in oceanic circulation, combined with sources of terrestrial carbon from the freshwater systems of the interior may require a different ΔR [69,70], but unfortunately, there are as yet no ΔR measurements for this region [71].

Many of the Beothuk dates produced calibrated age probability distributions that extend into the present, even though historical records indicate that some of the human remains were collected in the 19^th^ century. To help refine the radiocarbon age estimates we used a Bayesian modelling approach [13]. Our models treated both cultural groups as separate phases without assuming any chronological ordering within the dates. A posterior was applied for the end of the Beothuk time period using the historically documented year of Shanawdithit’s death (A.D. 1829) as the last known Beothuk [29]. This was applied to the Beothuk model as either a calendar date (OxCal CQL code function: C_date) *terminus ante quem*, or in the form of the OxCal constraint function for samples (n = 10) from contexts associated with modified European artifacts or trade items (e.g. worked iron, trade beads) were we assumed the correct calibrated date would fall between A.D. 1500 and A.D. 1829 ±10 years [66]. We tested for differences in the median calibrated dates between a Beothuk model run with and without a date constraint using a Wilcoxon Signed Rank test in SPSS v. 25. The Dorset age models used a uniformly distributed group boundary for the start and end of the phase, and included an estimate of the overall age span for the Dorset burials. Published dates from charcoal were included in both the Dorset and Beothuk models as outliers due to a lack of specific detail on the charcoal specimens dated and using a probability of 1 [66]. All OxCal Bayesian models were run a minimum of three times to test for robustness of the prior conditions and to determine the level of variation in the modelled ages. Typically, the modelled ages varied by +/- 5 years. Both the overall and individual sample date convergence and agreement of the models were at or above 100%. The OxCal CQL codes for the models analysed are included in S1 and S2 Text.

### Statistical and palaeodietary analysis

Statistical analyses of the isotope data were conducted using R v. 3.3.2 [72]. We applied an isotope Bayesian mixing model to assess the relative contributions of different groups of prey to Dorset and Beothuk diets. Potential prey species were grouped according to their δ^13^C and δ^15^N values using Kmeans cluster analysis. The optimal number of clusters was identified graphically using the Hubert and D- indices in the NbClust package [73]. As we were unable to sample archaeological salmon, we included carbon and nitrogen isotope data from the scales of adult Newfoundland salmon (n = 107) published by [74]. To account for the Suess Effect, the global depletion of ^13^C in the atmosphere due to the addition of fossil carbon [75], the salmon δ^13^C values were corrected by +1.5‰ which is in accordance with the estimated Suess Effect for the North Atlantic [76,77]. The δ^13^C values of sources were as follows: Cluster 1 = -15.8 ± 1.0‰; Cluster 2 = -11.8 ± 1.6‰; Cluster 3 = -19.7 ± 1.6‰; and Salmon = -14.9 ± 0.5‰. The δ^15^N values of the sources were: Cluster 1 = 15.1 ± 1.5‰; Cluster 2 = 16.4 ± 1.9‰; Cluster 3 = 4.2 ± 2.7‰; and Salmon = 11.5 ± 0.5‰. Human diets were analyzed using the SIAR [78] package in R using the siarmcmcderichletv4 function with 500000 iterations and a burnin of 50000. We applied a trophic enrichment factor (TEF) of 1.1 ± 0.2‰ and 5.5 ± 1.0‰ to the faunal carbon and nitrogen isotope values, respectively [79,80]. To compare Dorset and Beothuk diets and evaluate the impact of intensifying European settlement in the 18^th^ century, we defined three groups of human data using the median of the modelled calibrated radiocarbon dates and cultural/ethnohistoric information for each individual: Dorset, Beothuk pre-A.D. 1700, and Beothuk post-A.D. 1700 (or terminal). We did not include subadults in our statistical analyses. The human groups were further analyzed using one-way ANOVA followed by Dunnett’s T3 test for samples of unequal variance. We measured multiple skeletal elements for two individuals (NP 266 and XIII-A-1), but only included data from those bones representing a long-term average of diet (the skull and femur, respectively) in our statistical analyses.

## Results

### Radiocarbon dates, δ^13^C and δ^15^N values

Bone collagen was successfully extracted for both radiocarbon dating and palaeodietary analysis from all individuals. The complete list of stable isotope values, collagen quality indicators, and radiocarbon dates of the human samples in this study can be found in S2 and S3 Tables. Only those samples with acceptable collagen quality indicators were included in subsequent statistical analyses.

The isotope values of the faunal specimens, presented in S4 Table, were consistent with previously published data from subarctic species [3,4,81,82]. The δ^13^C and δ^15^N values of the aquatic fauna, which included a wide variety of species ranging from otters to harp seal to Great auks, averaged -14.8 ± 2.0‰ and 15.2 ± 1.6‰, respectively. Terrestrial fauna, which included caribou, beaver, black bear, and ptarmigan, among others, had mean δ^13^C and δ^15^N values of -19.9 ± 1.5‰ and 4.0 ± 2.7‰, respectively.

**Fig 2:**
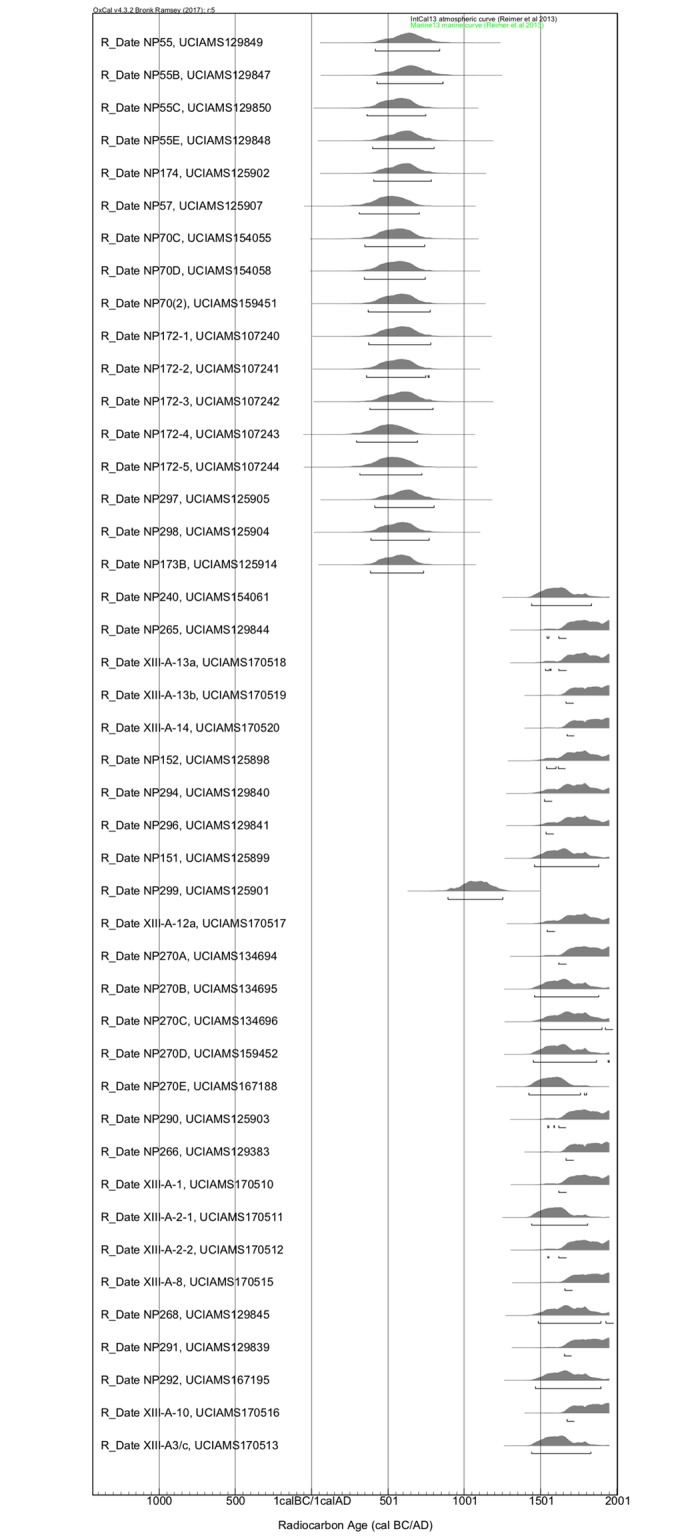
Modelled Dorset and Beothuk radiocarbon dates. Includes previously published dates from Raghavan et al. 2014 and Duggan et al. 2017.

The δ^13^C values of the 13 Dorset adults range from -14.1‰ to -12.5‰ with a mean of -13.1 ± 0.4‰. The δ^15^N values of the Dorset adults range from 19.7‰ to 21.5‰ with a mean of 20.6 ± 0.6‰ [7,12]. Our model of the 16 published [7,12] and new radiocarbon dates from Dorset individuals (including three subadults) (Fig 2) placed the dates within a 155 to 160-year (two-sigma) span of the Dorset occupation of Newfoundland, or between A.D. 400–600 (two-sigma) [18]. Our estimation of the carbon contributed from marine protein sources, determined through linear interpolation, exceeded 100% for 12 of the 16 dated Dorset bone samples, though given the uncertainty that we assigned to this estimation (±10%), and the uncertainty associated with the ΔR measurement (±50 years), it is likely that the true date falls within the calibrated two-sigma range of the ^14^C age. The radiocarbon and stable isotope results from the site of Englee provide further support to our hypothesis that this site is an authentic Dorset mortuary site distinct from that reported previously by [9]. The calibrated modelled radiocarbon dates fall between 390 and 600 cal AD, or within the Dorset occupation [18] and the high δ^13^C and δ^15^N values are consistent with those measured to date in other Dorset human remains from Newfoundland [12].

**Fig 3:**
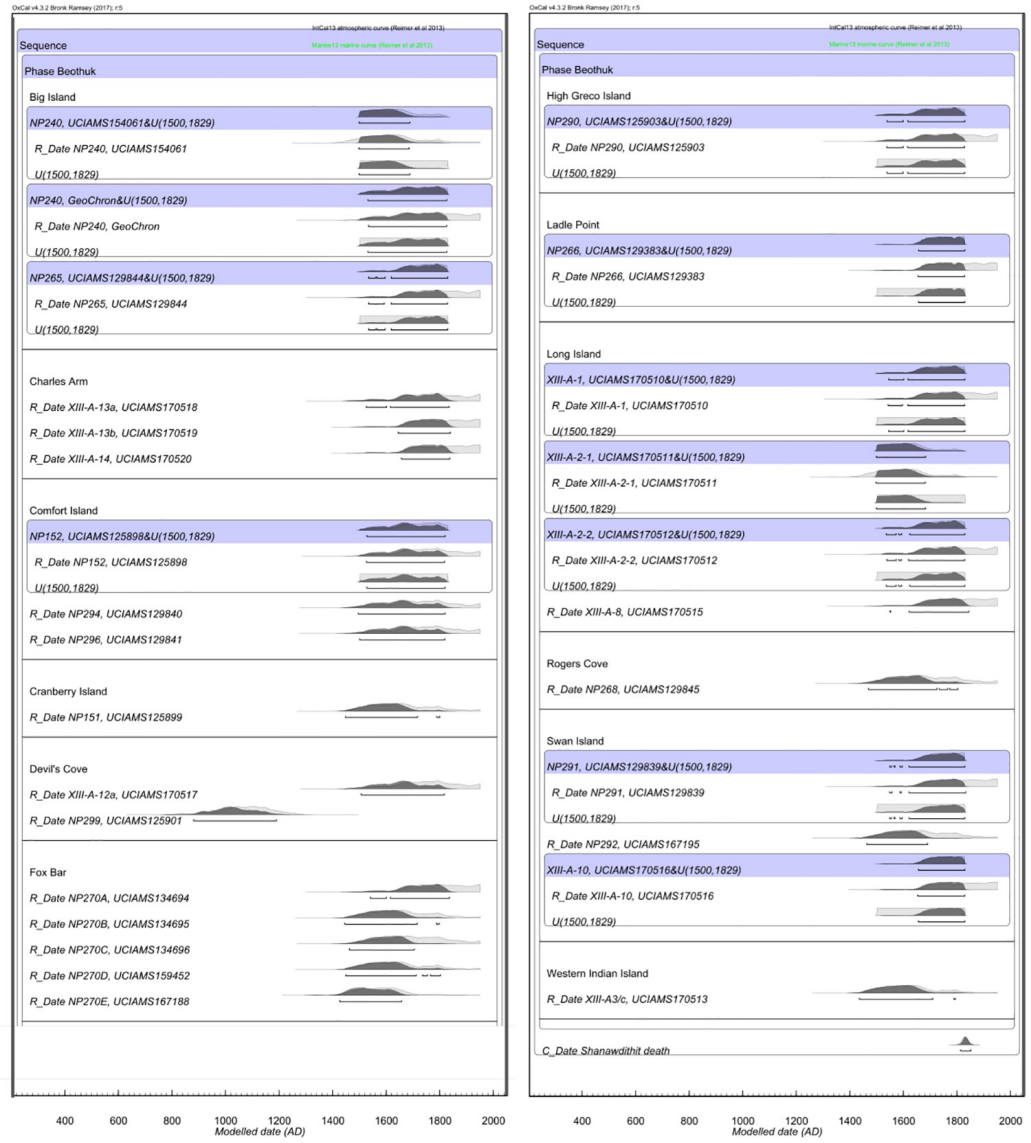
Calibrated Beothuk dates modelled with a date constraint of AD 1829. The purple shaded bars specify the parameters used to model the radiocarbon dates. Where the skeletal remains were found with modified European artifacts or trade items, the dates were constrained between AD 1500 and 1829. Includes published radiocarbon dates from Duggan et al. 2017.

The δ^13^C values of the 25 Beothuk adults range from -16.6‰ to -13.8‰ with a mean of -14.8 ± 1.4‰. The δ^15^N values range from 13.1‰ to 18.3‰, with a mean of 16.4 ± 1.4‰. The median calibrated radiocarbon dates are clustered in the historic period, with only one (NP 299) falling clearly within the pre-contact period with a calibrated two sigma range of AD 895–1255. Bayesian modelling of Beothuk dates using either the A.D. 1829 constraint only, or in combination with a start date of A.D. 1500 due to the presence of European-made burial offerings, such as harpoon heads or glass beads at certain sites, resulted in tighter probability distributions and significantly earlier calibrated dates than the modelling of the Beothuk dates without any constraint applied (*Z* = -4.631, *p* = 0.000) (Fig 3). The radiocarbon dates from five Beothuk sites revealed multiple chronologically distinct mortuary events. The dates from Big Island, Fox Bar, Long Island and Swan Island suggested separate periods of use in the 16^th^-17^th^ century and again in the 18^th^ century. The two individuals from Devil’s Cove dated to the 11^th^ century and 17^th^ century, however, given the poor archaeological context for this site, it is not clear if the recovered artifacts, such as the bone ornaments in particular, dated to the earlier or later period of use. The rib bone from the mummified subadult returned a calibrated date ranging between AD 1440–1695 (2 sigma), earlier than the date obtained previously from preserved soft tissue.

**Fig 4:**
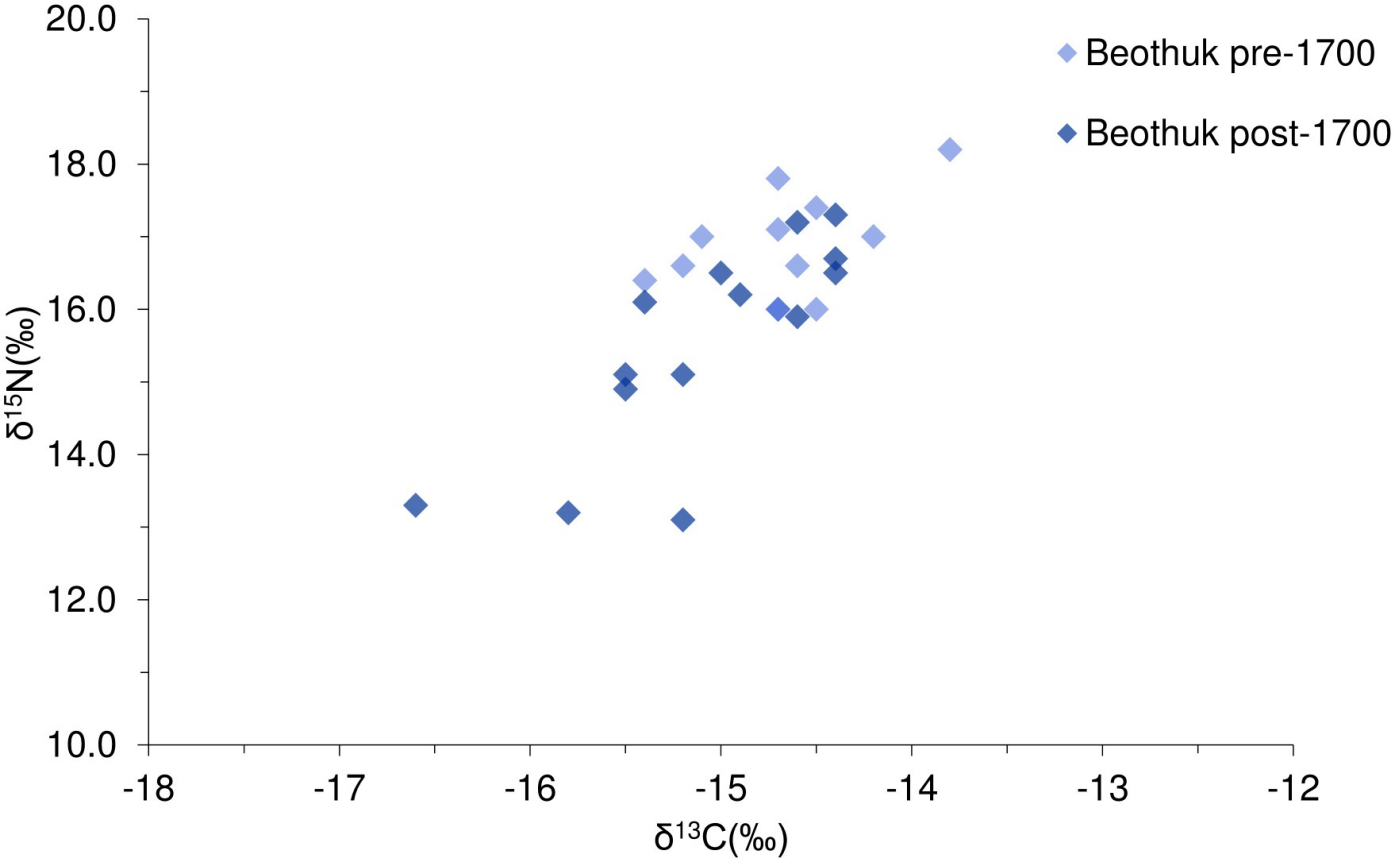
The δ^13^C and δ^15^N values of Beothuk individuals. The samples are separated chronologically by the median radiocarbon date of each sample. Includes published data from Duggan et al. 2017 and Kuch et al. 2007.

There is no apparent pattern between the geographic distribution of the burial sites and the stable isotope values of individuals buried at those sites. We noted a difference between the isotope values of individuals from the 18^th^ century and those dating to earlier time periods (Fig 4), however, this apparent change in dietary patterns is more evident between sites, than within sites with multiple mortuary events. We found significant differences between the δ^13^C values of all groups (*F* = 61.41, *p* = 0.000), but post hoc testing revealed that the difference could be attributed solely to the high δ^13^C values of the Dorset relative to the Beothuk, rather than to chronological differences in Beothuk diet (Table 2). Similarly, we also observed significant differences in the δ^15^N values of the cultural groups (*F* = 97.91, *p* = 0.000), but unlike the δ^13^C values, the diets of early pre-contact Beothuk were characterized by significantly higher δ^15^N values than those of the terminal Beothuk (Table 2). This suggests that the terminal, post-A.D. 1700 population consumed lower trophic level protein than those dating to an earlier period.

**Table 2:** Results of Dunnett’s T3 post hoc comparison between chronological groups.

Variable		Dorset (n = 13)	Beothuk < AD 1700 (n = 13)	Beothuk > AD 1700 (n = 15)
δ^13^C	Dorset	--	0.000	0.000
	Beothuk < 1700	0.000	--	0.141
δ^15^N	Dorset	--	0.000	0.000
	Beothuk < 1700	0.000	--	0.019

**Fig 5:**
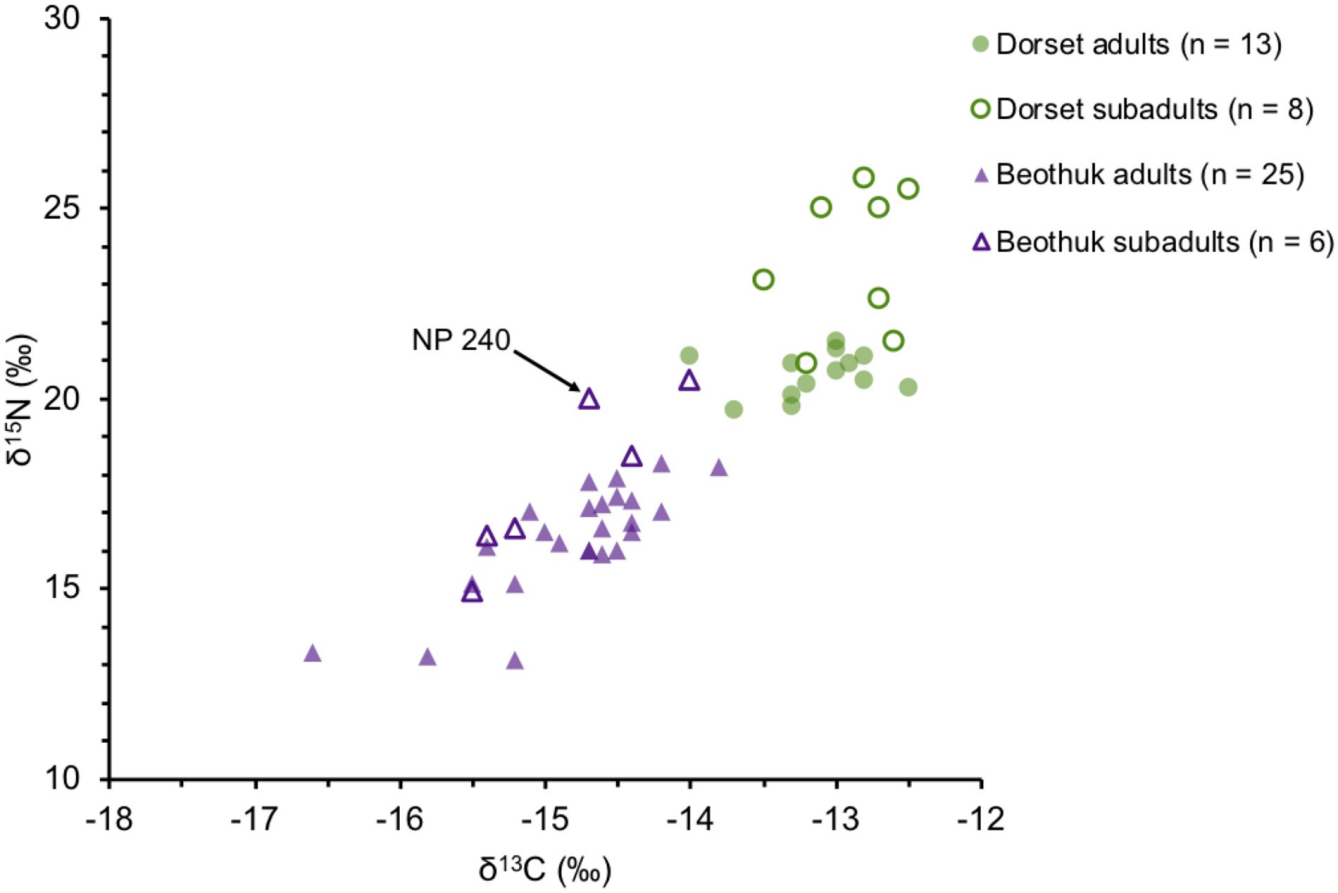
A comparison of the stable isotope values of Dorset and Beothuk adults and subadults. The naturally mummified subadult, NP 240, is indicated by an arrow. Includes published data from Duggan et al. 2017, Raghavan et al. 2014, and Kuch et al. 2007.

The δ^15^N values of five Dorset subadults and at least two Beothuk subadults were elevated relative to those of the adults from each respective cultural group, while six other subadults showed little to no isotopic offset that would be indicative of nursing [83] (Fig 5). The stable isotope values from the naturally mummified individual (NP 240) from Big Island indicate that this individual may not have been completely weaned at the time of death, although the unknown health status of the child complicates the interpretation of the δ^15^N values [84]. The radiocarbon date from NP 240 suggests that the child may have lived during the ‘Beothuk cultural fluorescence’, an unfortunately brief time period in the 17^th^ century that has been characterized by a more intensive Beothuk occupation of the northeast coast [23,28], however the δ^13^C value of the child is consistent with a generalized marine diet, suggesting that this period may not be associated with a marked change in the types of foods eaten. It is more difficult to interpret the stable isotope data from the other Beothuk subadults since many of their skeletal remains are incomplete, and/or commingled.

### Analysis of hunter-gatherer diets

**Fig 6:**
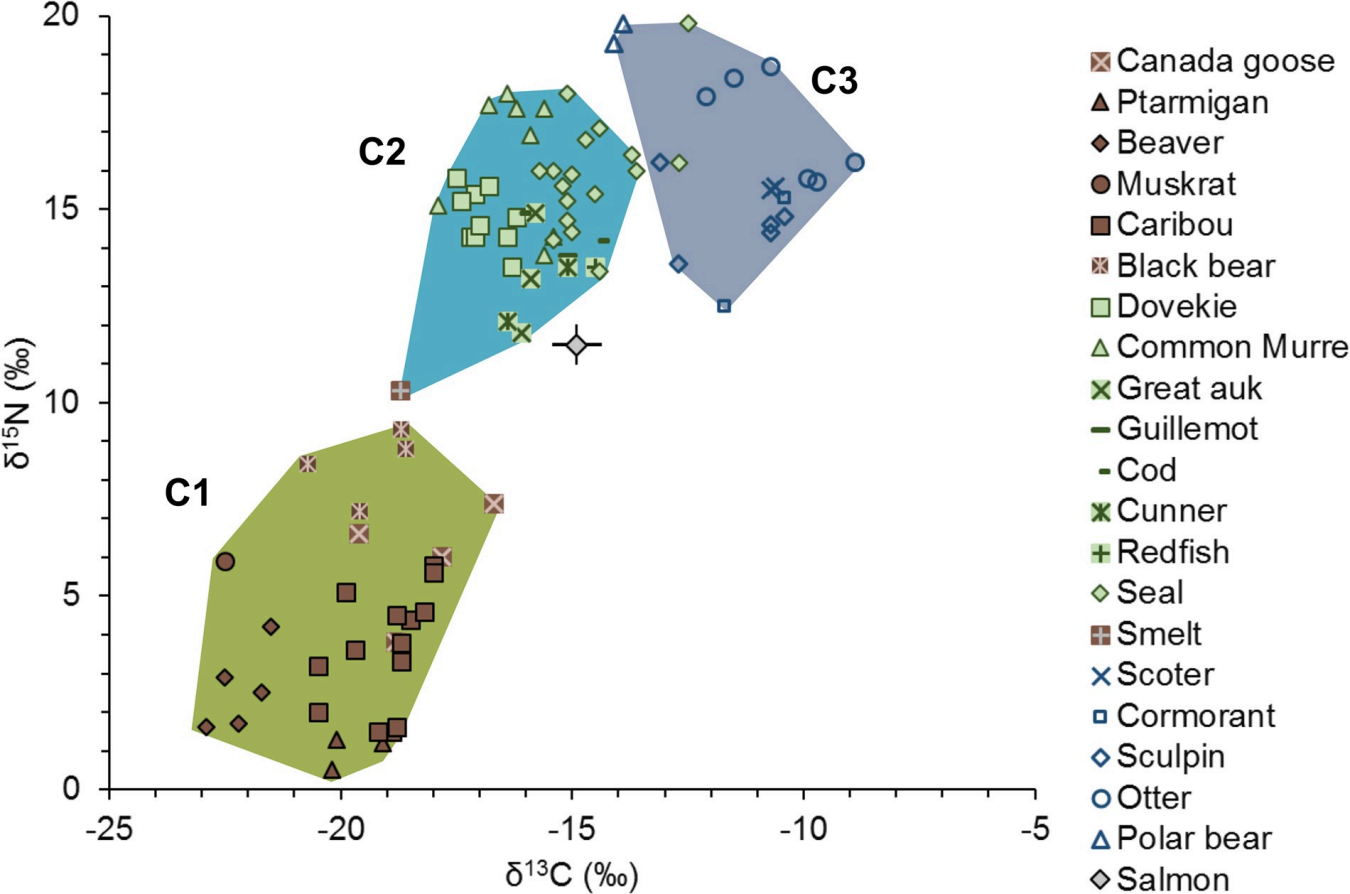
Clusters of archaeological fauna used in the SIAR model. The modern Newfoundland salmon data are from Dixon et al. 2012, and are corrected for the Suess Effect.

Statistical analysis of the archaeological faunal isotope values suggested that the data could be grouped into either two (marine and terrestrial) or three clusters of data (Fig 6) that roughly correspond to terrestrial species (C1) marine pelagic (C2), marine nearshore or benthic (C3) (Fig 5). The modern salmon data, corrected for the Suess Effect, plotted at the edge of the pelagic species which had potential confounding effects on the performance of the SIAR model [85].

**Fig 7:**
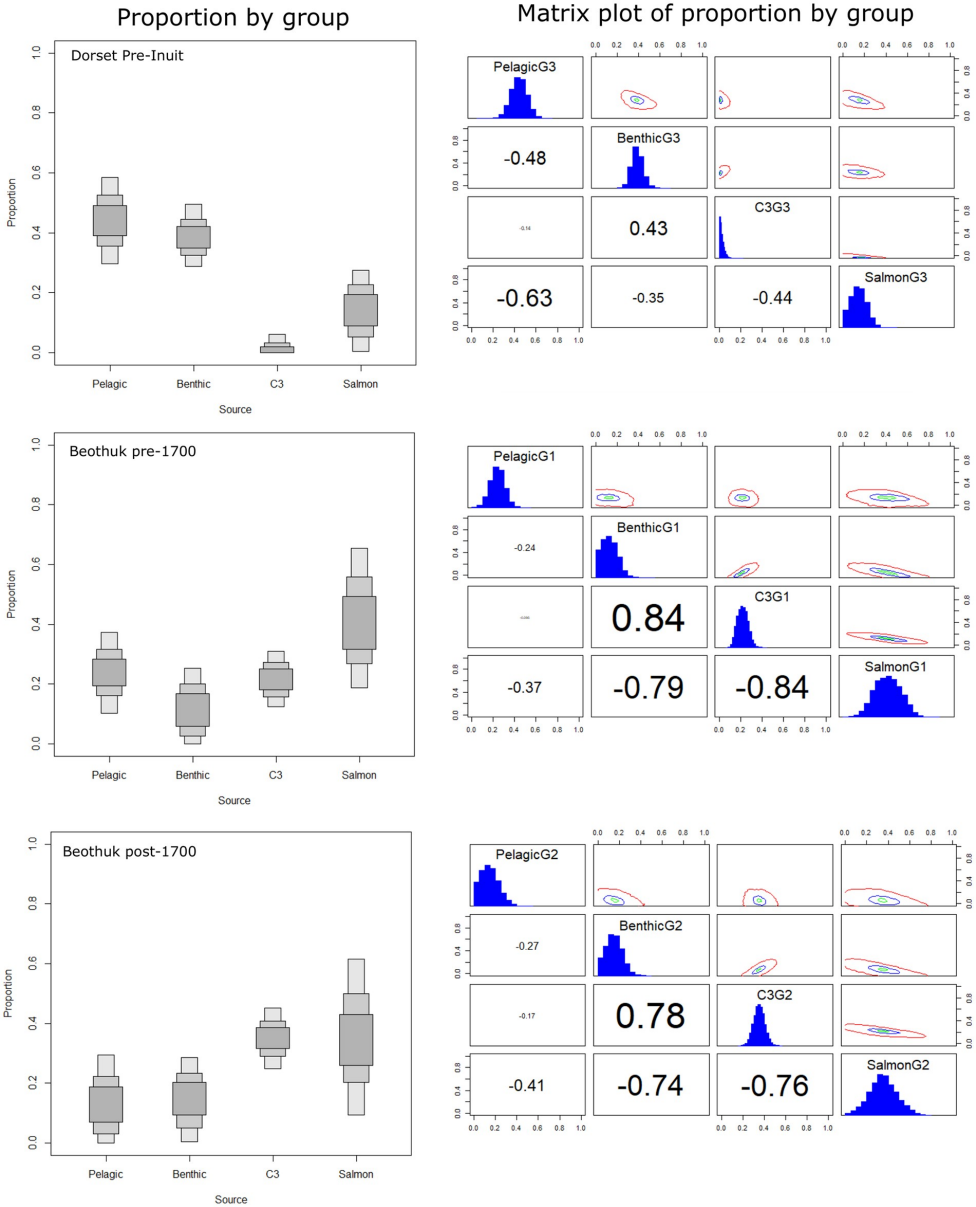
Estimated proportions of terrestrial (C1), pelagic (C2) and benthic (C3) food sources (left), and diagnostic matrix plots (right) for each cultural/chronological group. Model includes published human collagen isotope data from Raghavan et al. 2014, Duggan et al. 2017, and Kuch et al. 2007.

The results of the SIAR model are presented in Table 3 and Fig 7. Between 30 to 59% of Dorset dietary protein was obtained from a marine protein source consistent with the pelagic environment, while benthic or shallow water species provided between approximately 30 to 40% of dietary protein. A moderately negative correlation (-0.5) was associated with these sources indicating that both did not contribute equally to Dorset diet. Terrestrial species contributed between 0 and 6% to Dorset diet, while salmon may have contributed up to 30%. The residual errors associated with the Dorset δ^15^N values indicate that the model did not explain the high δ^15^N values of the Dorset relative to archaeological fauna, therefore the SIAR model results reported above should be interpreted with caution. This may have implications for our understanding of diet-bone collagen Δ^15^N offsets of high marine-protein consumers, like the Dorset and other specialized marine-adapted groups [42].

**Table 3:** Results of the SIAR model, including 95% credibility intervals, mode and median estimates.

Food sources	Low 95%	High 95%	Mode	Mean
*Dorset n = 13*				
Pelagic	0.30	0.59	0.44	0.44
Benthic	0.29	0.49	0.39	0.39
C_3_ fauna	0.00	0.06	0.01	0.02
Salmon	0.00	0.28	0.14	0.15
SD δ^13^C	0.00	0.57	0.05	0.22
SD δ^15^N	0.00	1.10	0.09	0.44
*Beothuk pre-AD 1700 n = 13*				
Pelagic	0.10	0.37	0.24	0.24
Benthic	0.00	0.25	0.12	0.13
C_3_ fauna	0.13	0.31	0.22	0.22
Salmon	0.19	0.65	0.41	0.42
SD δ^13^C	0.00	0.53	0.04	0.21
SD δ^15^N	0.00	0.91	0.08	0.36
*Beothuk post-AD 1700 n = 15*				
Pelagic	0.00	0.29	0.13	0.15
Benthic	0.00	0.29	0.15	0.15
C_3_ fauna	0.25	0.45	0.34	0.35
Salmon	0.09	0.62	0.33	0.35
SD δ^13^C	0.00	0.71	0.11	0.30
SD δ^15^N	0.00	1.82	0.84	0.90

SD is the residual error associated with carbon or nitrogen for each cultural group. Model includes published human collagen isotope data from Raghavan et al. 2014, Duggan et al. 2017, and Kuch et al. 2007.

The diets of pre- AD 1700 Beothuk may have been largely composed of salmon (~20–65%), with moderate contributions from marine and terrestrial sources of protein. We noted a strong negative correlation between salmon and benthic species (-0.8) and between salmon and terrestrial protein (-0.8) indicating that these sources could not have made equal contributions to Beothuk diet. The pattern of declining δ^15^N values in the historic Beothuk population was apparent in the SIAR model results. We observed a decrease in the contribution from the pelagic species (0 - ~30%), and an increase in the consumption of terrestrial species (25–45%). It should be noted that the residual errors associated with the δ^13^C and δ^15^N values of the pre-AD 1700 and terminal Beothuk were high, which may be due to the large standard deviations of each faunal group, the variation within each chronological Beothuk group, or a missing dietary source, such as freshwater fish.

## Discussion

### Hunter-gatherer subsistence practices in Newfoundland

Through the analysis of stable carbon and nitrogen isotope ratios measured in the bone collagen of humans and animals, the diets of Newfoundland hunter-gatherers appear to be distinct and the isotopic variation can be attributed to cultural and chronological differences in food preference and procurement. These data are significant because they allow the relationship of settlement patterns, diet and human skeletal chemistry to be examined. Here we will discuss the isotope values of each cultural group in relation to the radiocarbon chronology, and in light of archaeological research on the island of Newfoundland.

The isotope values of the Dorset are indicative of a highly specialized marine adaptation, and with respect to settlement patterns, support the hypothesis that the Dorset preferentially selected outer coasts and islands, such as the Port au Choix area, to settle. Based on the results of the SIAR model we suggest that the Dorset hunted both pelagic species and those occupying the nearshore regions of the island. With few exceptions [86,87], Dorset faunal assemblages are overwhelmingly composed of seal remains, thus it is possible that, in addition to specializing in the harp seal hunt, the Dorset also targeted species such as the harbour seal.

The isotope data from human skeletons affiliated with the Beothuk culture suggests that their diets featured a wider range of protein sources, as indicated by more variable δ^13^C and δ^15^N values. One individual (NP 299) dates to the pre-European contact period and has δ^13^C and δ^15^N values that are suggestive of a generalized, but predominantly marine diet, similar to the Beothuk from the early historic period. At this point in Newfoundland history, the island was undergoing profound cultural change with the departure of the Dorset, and the gradual integration of small projectile points, associated with bow-and-arrow technology into the toolkits of precontact Beothuk archaeological complexes. This may have facilitated the development of a settlement system that gave the Beothuk greater flexibility in the subarctic environment [88].

### European colonization and Beothuk lifeways

We gain significant insight into the diets of the Beothuk people by examining the variation in their isotope values relative to the chronology of post-European contact Newfoundland. Previous analyses of Beothuk site distribution and excavated Beothuk faunal assemblages would suggest that, unlike the Dorset, the Beothuk were not a specialized seal-hunting culture. Beothuk diets appear isotopically consistent with a generalized marine-adaptation with the greatest inputs of protein sourced from the salmon fishery, and from pelagic species. The pelagic species likely included seal, but the contribution from isotopically similar seabirds should not be overlooked [89]. It is interesting to note that the individuals with the highest δ^13^C and δ^15^N values were those buried at the site of Fox Bar (DeAk-2), on the coast of Bonavista Bay. The nearby Beaches archaeological site (DeAk-1) was identified as a probable launching point for a Beothuk harp seal hunt [17] as the site lies near one of the breeding territories of the harp seal [90]. Although land use practices are not well understood with respect to the mobility of Beothuk families [24], this may be evidence that proximity to the territory in which they lived was a consideration for choice of burial site (see also [89]). The considerable contribution of marine and anadromous protein to pre-AD 1700 Beothuk diet is significant in relation to the archaeological record for this time period. The 17^th^ -18^th^ century sites of Boyd’s Cove and Inspector Island on the northeastern coast, and the Beaches site on the east coast of the island may evidence a period of cultural fluorescence that stemmed from access to European goods, such as iron nails and sails, granted by the migratory character of the European fishery [23]. Coastal Beothuk sites during this time period become larger with more substantial dwellings and midden features than pre-contact sites [28]. The significance of salmon to Beothuk diet is further supported by the inclusion of a pouch of dried salmon in the internment of the mummified child from Big Island [29]. Although the isotope data are thus not unexpected, they emphasize the important role that coastal and riverine resources played in Beothuk subsistence.

It appears from the isotopic data that with the onset of the 18^th^ century, the traditional foodways of the Beothuk underwent a marked change, although the abandonment of the coast was not complete. The δ^15^N values from skeletal remains post-dating AD 1700 are significantly lower than those dating to earlier centuries, but as the dated skeletal material shows, this trend is not consistent between burial sites. For example, while the individuals buried at Charles’ Arm, Ladle Point, and Red Indian Lake have lower δ^15^N values that would be consistent with a greater reliance on terrestrial sources of protein, broadly contemporaneous individuals from Fox Bar, and Long Island have δ^15^N values that are consistent with the earlier Beothuk diet. This suggests regional differences in the effects of European settlement on Beothuk lifeways, perhaps depending on settlement intensity of both groups, and differential resource distribution [24].

Evidence of dietary change can also be found in the archaeological record: Few archaeological sites dating to the late 18^th^ to early 19^th^ century (burial sites excepted) have been found on the coast while larger sites with more substantial pit-style dwellings, middens of caribou bone, and evidence for storage structures have been found in the interior of the island and indicate an attempt to maximize economic returns from the caribou hunt, perhaps to enable survival in the interior for a greater part of the year [28,91]. These efforts may be most observable in the isotope values from the Red Indian Lake and Ladle Point individuals. Without the benefit of faunal data, or an understanding of the range of isotopic variation in other Beothuk people, previous authors [11] presumed that the isotope values from the dentinal collagen of the Beothuk chief, Nonosabasut, and his wife, Demasduit, resulted from a regular consumption of marine and anadromous species. Instead, when the isotope values of Nonosabasut and Demasduit are compared to those of other Beothuk people, and to local archaeological fauna, their diets are considerably more terrestrial, and represent a striking deviation from the foodways that characterized the Beothuk of the past. This is even more apparent in the pair of δ^13^C and δ^15^N values from the Ladle Point individual’s second molar and skull bone. The small negative shift in excess of analytical error in the δ^13^C and δ^15^N values between the second molar, which reflects diet during childhood, and the skull bone, which should reflect long term diet, points to a small decrease in the amount of marine protein consumed by the Ladle Point individual. Therefore, the bioarcheological data presented here, and by [7,11], support the archaeological record, and, given the paucity of human remains directly affiliated with the Beothuk culture, these data offer a glimpse of the impact that European settlement had on the Beothuk way of life.

## Conclusions

The isotopic results from the analysis of human and faunal remains affiliated with the Dorset and the Beothuk cultures served to better characterize the diets of Newfoundland’s hunter-gatherers and validated current hypotheses of human subsistence rooted in ethnohistoric and archaeological research. The difference between Dorset and Beothuk settlement patterns identified archaeologically is reflected in the bioarchaeological data from skeletal remains affiliated with these cultures and can be attributed to real differences in subsistence practices. The Dorset located their settlements in the outer coastal regions of the island for the purpose of accessing migrating and local seal herds, while the Beothuk people employed a stable, generalized-marine adaptation that encompassed a wide range of predominantly marine species, but may have included terrestrial species as well. Our data show a marked change in Beothuk diet after the 18^th^ century that likely corresponded to the withdrawal of the Beothuk from the coast in response to the presence of European settlers. We further noted some geographic variation that may relate to intensity of contact with Europeans. The stable isotope values from Beothuk after the 18^th^ century indicate that while the Beothuk were able to access some marine foods, the general composition of the diet changed with implications for maintenance of traditional foodways.

## Supporting information

S1 Table Isotopic standards. (XLSX)

S2 Table Human stable isotope values. (XLSX)

S3 Table Human radiocarbon dates and model ages. (XLSX)

S4 Table Faunal stable isotope values. (XLSX)

S1 Text Oxcal CQL code_Dorset dates. (DOCX)

S2 Text Oxcal CQL code_Beothuk dates. (DOCX)
