# Supplementary material for: Dorset Pre-Inuit and Beothuk foodways in Newfoundland, ca. AD 500-1829
Source: PLoS One. 2019 Jan 7;14(1):e0210187. doi: 10.1371/journal.pone.0210187 (PMC6322756; doi:10.1371/journal.pone.0210187)
Supplement: S1 Text — (DOCX) [file pone.0210187.s005.docx]

Oxcal CQL Codes for Dorset Human dates

File: NF_Human_Chrono/Dorset_Phase_Sites_1.oxcal

Plot()

{

Curve("IntCal13", "IntCal13.14c");

Curve("Marine13", "Marine13.14c");

// Delta_R for Labrador Sea is 140+/-50 (Raghavan et al. 2014, Science 345(6200):1255832-1 - 1255832-9).

Delta_R("LocalMarine", 140, 50);

Sequence()

{

Boundary("start");

Phase("Dorset")

{

Line();

Label("Crow Head Cave");

Mix_Curves("Mix NP55", "IntCal13", "LocalMarine", 110, 15);

R_Date("NP55, UCIAMS129849", 1955, 25);

Mix_Curves("Mix NP55B", "IntCal13", "LocalMarine", 115, 15);

R_Date("NP55B, UCIAMS129847", 1965, 20);

Mix_Curves("Mix NP55C", "IntCal13", "LocalMarine", 96, 15);

R_Date("NP55C, UCIAMS129850", 1950, 25);

Mix_Curves("Mix NP55E", "IntCal13", "LocalMarine", 106, 15);

R_Date("NP55E, UCIAMS129848", 1960, 25);

Line();

Label("Eastern Point");

Mix_Curves("Mix NP174", "IntCal13", "LocalMarine", 100, 15);

R_Date("NP174, UCIAMS125902", 1935, 15);

Line();

Label("Englee/Lane's Cove");

Mix_Curves("Mix NP57", "IntCal13", "LocalMarine", 102, 15);

R_Date("NP57, UCIAMS125907", 2030, 15);

Mix_Curves("Mix NP70C", "IntCal13", "LocalMarine", 106, 15);

R_Date("NP70C, UCIAMS154055", 2010, 15);

Mix_Curves("Mix NP70D", "IntCal13", "LocalMarine", 110, 15);

R_Date("NP70D, UCIAMS154058", 2030, 15);

Mix_Curves("Mix NP70(2)", "IntCal13", "LocalMarine", 106, 15);

R_Date("NP70(2), UCIAMS159451", 1995, 25);

Line();

Label("Gargamelle Rockshelter");

Mix_Curves("Mix NP172-1", "IntCal13", "LocalMarine", 110, 15);

R_Date("NP172-1, UCIAMS107240", 2010, 20);

Mix_Curves("Mix NP172-2", "IntCal13", "LocalMarine", 106, 15);

R_Date("NP172-2, UCIAMS107241", 2000, 15);

Mix_Curves("Mix NP172-3", "IntCal13", "LocalMarine", 115, 15);

R_Date("NP172-3, UCIAMS107242", 2020, 15);

Mix_Curves("Mix NP172-4", "IntCal13", "LocalMarine", 100, 15);

R_Date("NP172-4, UCIAMS107243", 2030, 15);

Mix_Curves("Mix NP172-5", "IntCal13", "LocalMarine", 108, 15);

R_Date("NP172-5, UCIAMS107244", 2050, 15);

Line();

Label("Indian Cove");

Mix_Curves("Mix NP297", "IntCal13", "LocalMarine", 102, 15);

R_Date("NP297, UCIAMS125905", 1930, 15);

Mix_Curves("Mix NP298", "IntCal13", "LocalMarine", 100, 15);

R_Date("NP298, UCIAMS125904", 1960, 20);

Line();

Label("Phillip's Garden");

Mix_Curves("Mix NP173B", "IntCal13", "LocalMarine", 87, 15);

R_Date("NP173B, UCIAMS125914", 1905, 15);

Line();

};

Boundary("end");

Span("Dorset");

};

};
