# Supplementary material for: Dorset Pre-Inuit and Beothuk foodways in Newfoundland, ca. AD 500-1829
Source: PLoS One. 2019 Jan 7;14(1):e0210187. doi: 10.1371/journal.pone.0210187 (PMC6322756; doi:10.1371/journal.pone.0210187)
Supplement: S2 Text — (DOCX) [file pone.0210187.s006.docx]

File: NF_Human_Chrono/Beothuk_Sites_7_wConstrain_wNP299.oxcal

CQL Code for model:

Plot ()

{

Curve("IntCal13", "IntCal13.14c");

Curve("Marine13", "Marine13.14c");

// Delta_R for Labrador Sea is 140+/-50 (Raghavan et al. 2014, Science 345(6200):1255832-1 - 1255832-9).

Delta_R("LocalMarine", 140, 50);

Sequence()

{

Phase("Beothuk")

{

Label("Big Island");

Mix_Curves("Mix NP240a", "IntCal13", "LocalMarine", 73, 15);

R_Date("NP240, UCIAMS154061", 675, 20) & Date(U(1500,1829));

Mix_Curves("Mix NP240b", "IntCal13", "LocalMarine", 73, 15);

R_Date("NP240, GeoChron", 549, 62) & Date(U(1500,1829));

Mix_Curves("Mix NP265", "IntCal13", "LocalMarine", 67, 15);

R_Date("NP265, UCIAMS129844", 500, 25) & Date(U(1500,1829));

Line();

Label("Charles Arm");

Mix_Curves("Mix XIII-A-13a", "IntCal13", "LocalMarine", 60, 15);

R_Date("XIII-A-13a, UCIAMS170518", 480, 15);

Mix_Curves("Mix XIII-A-13b", "IntCal13", "LocalMarine", 58, 15);

R_Date("XIII-A-13b, UCIAMS170519", 415, 15);

Mix_Curves("Mix XIII-A-14", "IntCal13", "LocalMarine", 58, 15);

R_Date("XIII-A-14, UCIAMS170520", 405, 15);

Line();

Label("Comfort Island");

Mix_Curves("Mix NP152", "IntCal13", "LocalMarine", 77, 15);

R_Date("NP152, UCIAMS125898", 590, 15) & Date(U(1500,1829));

Mix_Curves("Mix NP294", "IntCal13", "LocalMarine", 73, 15);

R_Date("NP294, UCIAMS129840", 590, 25);

Mix_Curves("Mix NP296", "IntCal13", "LocalMarine", 79, 15);

R_Date("NP296, UCIAMS129841", 610, 25);

Line();

Label("Cranberry Island");

Mix_Curves("Mix NP151", "IntCal13", "LocalMarine", 75, 15);

R_Date("NP151, UCIAMS125899", 655, 15);

Line();

Label("Devil's Cove");

Mix_Curves("Mix XIII-A-12a", "IntCal13", "LocalMarine", 83, 15);

R_Date("XIII-A-12a, UCIAMS170517", 625, 20);

Mix_Curves("Mix NP299", "IntCal13", "LocalMarine", 73, 15);

R_Date("NP299, UCIAMS125901", 1335, 15);

Line();

Label("Fox Bar");

Mix_Curves("Mix NP270A", "IntCal13", "LocalMarine", 79, 15);

R_Date("NP270A, UCIAMS134694", 555, 20);

Mix_Curves("Mix NP270B", "IntCal13", "LocalMarine", 77, 15);

R_Date("NP270B, UCIAMS134695", 665, 20);

Mix_Curves("Mix NP270C", "IntCal13", "LocalMarine", 90, 15);

R_Date("NP270C, UCIAMS134696", 700, 20);

Mix_Curves("Mix NP270D", "IntCal13", "LocalMarine", 62, 15);

R_Date("NP270D, UCIAMS159452", 595, 25);

Mix_Curves("Mix NP270E", "IntCal13", "LocalMarine", 79, 15);

R_Date("NP270E, UCIAMS167188", 755, 15);

Line();

Label("High Greco Island");

Mix_Curves("Mix NP290", "IntCal13", "LocalMarine", 75, 15);

R_Date("NP290, UCIAMS125903", 545, 15) & Date(U(1500,1829));

Line();

Label("Ladle Point");

Mix_Curves("Mix NP266", "IntCal13", "LocalMarine", 39, 15);

R_Date("NP266, UCIAMS129383", 315, 25) & Date(U(1500,1829));

Line();

Label("Long Island");

Mix_Curves("Mix XIII-A-1", "IntCal13", "LocalMarine", 75, 15);

R_Date("XIII-A-1, UCIAMS170510", 540, 15) & Date(U(1500,1829));

Mix_Curves("Mix XIII-A-2-1", "IntCal13", "LocalMarine", 63, 15);

R_Date("XIII-A-2-1, UCIAMS170511", 640, 15) & Date(U(1500,1829));

Mix_Curves("Mix XIII-A-2-2", "IntCal13", "LocalMarine", 69, 15);

R_Date("XIII-A-2-2, UCIAMS170512", 510, 15) & Date(U(1500,1829));

Mix_Curves("Mix XIII-A-8", "IntCal13", "LocalMarine", 79, 15);

R_Date("XIII-A-8, UCIAMS170515", 525, 20);

Line();

Label("Rogers Cove");

Mix_Curves("Mix NP268", "IntCal13", "LocalMarine", 77, 15);

R_Date("NP268, UCIAMS129845", 640, 15);

Line();

Label("Swan Island");

Mix_Curves("Mix NP291", "IntCal13", "LocalMarine", 79, 15);

R_Date("NP291, UCIAMS129839", 530, 25) & Date(U(1500,1829));

Mix_Curves("Mix NP292", "IntCal13", "LocalMarine", 87, 15);

R_Date("NP292, UCIAMS167195", 710, 15);

Mix_Curves("Mix XIII-A-10", "IntCal13", "LocalMarine", 63, 15);

R_Date("XIII-A-10, UCIAMS170516", 430, 15) & Date(U(1500,1829));

Line();

Label("Western Indian Island");

Mix_Curves("Mix XIII-A3/c", "IntCal13", "LocalMarine", 65, 15);

R_Date("XIII-A3/c, UCIAMS170513", 630, 15);

Line();

};

C_date("Shanawdithit death", 1829,10);

};

};
